# The COPD risk associated with adulthood weight change and early adulthood BMI: a prospective cohort study

**DOI:** 10.1186/s12889-025-25582-z

**Published:** 2025-11-22

**Authors:** Lu Chen, Wei Yu, Dianjianyi Sun, Pei Pei, Ling Yang, Yiping Chen, Huaidong Du, Xujun Yang, Maxim Barnard, Junshi Chen, Zhengming Chen, Jun Lv, Liming Li, Canqing Yu, China Kadoorie Biobank Collaborative Group, Robert Clarke, Rory Collins, Richard Peto, Robin Walters, Daniel Avery, Derrick Bennett, Ruth Boxall, Jonathan Clarke, Ahmed Edris Mohamed, Hannah Fry, Yani Huang, Pek Kei Im, Andri Iona, Christiana Kartsonaki, Kshitij Kolhe, Hubert Lam, Kuang Lin, James Liu, Iona Millwood, Sam Morris, Qunhua Nie, Alfred Pozarickij, Maryam Rahmati, Paul Ryder, Maruf Sarder, Dan Schmidt, Becky Stevens, Baihan Wang, Lin Wang, Neil Wright, Xiaoming Yang, Pang Yao, Xiao Han, Can Hou, Qingmei Xia, Chao Liu, Lang Pan, Zengchang Pang, Ruqin Gao, Shanpeng Li, Haiping Duan, Shaojie Wang, Yongmei Liu, Ranran Du, Yajing Zang, Liang Cheng, Xiaocao Tian, Hua Zhang, Yaoming Zhai, Feng Ning, Xiaohui Sun, Feifei Li, Silu Lv, Junzheng Wang, Wei Hou, Wei Sun, Shichun Yan, Xiaoming Cui, Chi Wang, Zhenyuan Wu, Yanjie Li, Quan Kang, Huiming Luo, Tingting Ou, Xiangyang Zheng, Zhendong Guo, Shukuan Wu, Yilei Li, Huimei Li, Ming Wu, Yonglin Zhou, Jinyi Zhou, Ran Tao, Jie Yang, Jian Su, Fang Liu, Jun Zhang, Yihe Hu, Yan Lu, Liangcai Ma, Aiyu Tang, Shuo Zhang, Jianrong Jin, Jingchao Liu, Mei Lin, Zhenzhen Lu, Lifang Zhou, Changping Xie, Jian Lan, Tingping Zhu, Yun Liu, Liuping Wei, Liyuan Zhou, Ningyu Chen, Yulu Qin, Sisi Wang, Xianping Wu, Ningmei Zhang, Xiaofang Chen, Xiaoyu Chang, Mingqiang Yuan, Xia Wu, Wei Jiang, Jiaqiu Liu, Qiang Sun, Faqing Chen, Xiaolan Ren, Caixia Dong, Hui Zhang, Enke Mao, Xiaoping Wang, Tao Wang, Xi zhang, Kai Kang, Shixian Feng, Huizi Tian, Lei Fan, XiaoLin Li, Huarong Sun, Pan He, Xukui Zhang, Min Yu, Ruying Hu, Hao Wang, Xiaoyi Zhang, Yuan Cao, Kaixu Xie, Lingli Chen, Dun Shen, Xiaojun Li, Donghui Jin, Li Yin, Huilin Liu, Zhongxi Fu, Xin Xu, Hao Zhang, Jianwei Chen, Yuan Peng, Libo Zhang, Chan Qu

**Affiliations:** 1 Department of Epidemiology and Biostatistics, School of Public Health, Peking University, 38 Xueyuan Road, Beijing, 100191 China; 2 Peking University Center for Public Health and Epidemic Preparedness & Response, Beijing, 100191 China; 3 Key Laboratory of Epidemiology of Major Diseases (Peking University), Ministry of Education, Beijing, 100191 China; 4 Clinical Trial Service Unit & Epidemiological Studies Unit (CTSU), Nuffield Department of Population Health, University of Oxford, Oxford, OX3 7LF UK; 5 Maiji Center for Disease Control and Prevention, Maiji, 741020 China; 6 China National Center for Food Safety Risk Assessment, Beijing, 100022 China; 7 State Key Laboratory of Vascular Homeostasis and Remodeling, Peking University, Beijing, 100191 China

**Keywords:** Chronic obstructive pulmonary disease, Weight change, Body mass index, Early adulthood

## Abstract

**Background:**

Gaps concerning general adiposity and chronic obstructive pulmonary disease (COPD) are delaying policies for weight interventions in COPD prevention, especially adiposity in early adulthood and weight changes during adulthood.

**Methods:**

Based on the China Kadoorie Biobank, a prospective cohort between 2004–2008 covering 5 urban and 5 rural areas, we included 138,764 males and 194,159 females aged 35 ~ 70 years. Weight change was defined as the difference between directly measured weight at baseline and self-reported weight at age 25. Incident COPD events were followed-up until the end of 2018. Cox proportional hazard models were conducted to estimate the hazard ratios (HRs) and 95% confidence intervals (CIs) for associations of adulthood weight change and early adulthood body mass index (BMI) with COPD risk.

**Results:**

During a mean follow-up of 11.9 years, 3,732 and 3,154 incident COPD events occurred in males and females, respectively. In males, weight change and early adulthood BMI were inversely associated with COPD risk, while early adulthood obesity (BMI ≥ 28.0 kg/m^2^) was associated with an increased risk of COPD (HR = 1.76; 95% CI: 1.01, 3.08), compared with the 18.5 ~ 23.9 kg/m^2^ early adulthood BMI group, among non-current smokers. In females, extreme weight loss and gain were associated with increased COPD risk. The effects of weight loss and gain on COPD were enhanced in the underweight and obese population, respectively, in early adulthood.

**Conclusions:**

Weight change and early adulthood BMI were inversely linked to COPD risk in males, while in females, both extreme weight loss or gain increased COPD risk.

**Supplementary Information:**

The online version contains supplementary material available at 10.1186/s12889-025-25582-z.

## Introduction

With economic development, accompanied by shifts in dietary patterns and the adoption of sedentary lifestyles [1], the global prevalence of obesity has exhibited a marked trend of getting higher and younger. Early adulthood represents a critical juncture for weight gain, during which many individuals experience an accumulation of excess adiposity [2]. This period is often characterized by significant life-course transitions, like childbirth, which may presage future obesity [3]. Studies have found that overweight and obesity in early adulthood and weight changes during adulthood are associated with a range of adverse health outcomes, including cardiometabolic diseases [4–6], digestive tract cancer^7^, and breast cancer [2]. Despite these associations, weight management during early adulthood is frequently overlooked, as the health implications of modest weight gain may not yet be sufficiently pronounced to prompt medical consultation [3].

In 2019, chronic obstructive pulmonary disease (COPD) ranked third in the cause of death both globally and in China, imposing a substantial burden on public health [7]. Previous research has yielded inconsistent conclusions concerning general adiposity and COPD as well as lung function [8–10]. Gaps are delaying public health policies for weight interventions in COPD prevention. To our knowledge, no studies have focused on the associations of adiposity in early adulthood and weight changes during adulthood with the risk of COPD. Nonetheless, existing literature indicates that the duration of overweight or obesity is an independent risk factor for impaired lung function [11], and being overweight or obese in early adulthood means prolonged exposure to obesity. Clarifying the association of general adiposity in early adulthood and adulthood weight changes with the risk of COPD bears great public health significance. Such exploration is crucial for a obtaining comprehensive understanding of the impact of obesity, controlling of obesity-related chronic diseases, and promoting of population health.

Based on the China Kadoorie Biobank (CKB) study of middle-aged and elderly individuals, we investigated associations of weight change since early adulthood and early adulthood body mass index (BMI) with COPD risk. Given the sex heterogeneity in weight and COPD, all associations were independently examined in males and females.

## Methods

### Study population

The design of CKB has been described extensively elsewhere [12, 13]. Briefly, a community-based cohort recruiting ~ 500,000 participants from five urban and five rural areas in China was established between 2004 and 2008. Among the 470,028 participants aged 35 ~ 70 years, we excluded those with self-reported or screened COPD (*n* = 30,638) and other self-reported major chronic diseases (diabetes, heart disease, stroke, cancer, asthma, and tuberculosis; *n* = 46,625), missing baseline values of weight (*n* = 1) and self-reported weight at age 25 (*n* = 59,569), and implausible baseline forced expiratory volume in one second (FEV1) and forced vital capacity (FVC) values (FEV1/FVC > 1, *n* = 272). Finally, 138,764 males and 194,159 females remained in the primary analysis.

### Assessment of weight and height

The middle or late adulthood weight and height were directly measured via calibrated gauges by trained technicians at the baseline survey. Early adulthood weight data were collected based on self-reports by participants through reflections on their weight at approximately age 25 years. The reported early adulthood weight obtained during baseline was consistent with that recorded at the first resurvey in 2008 among the 11,602 participants who took part in both surveys, with an Pearson’s correlation coefficient of 0·81. The weight change was calculated as the difference between the weight at baseline and the weight around age 25 years.

BMI is an index that calculates weight for height. Early adulthood BMI was computed as the ratio of weight (kg) at approximately age 25 years to baseline height (m) squared.

### Assessment of covariates and lung function

Age, sex, and region were determined according to the household registry and identity number. Additional sociodemographic information, including household income, occupation, education and marital status, was gathered by interviewer-administered questionnaires, along with lifestyle factors [14]. Among ever-smokers, we collected data on the type, frequency, and daily amount of tobacco consumption, as well as the age at smoking initiation. The duration of smoking was calculated by subtracting the age at initiation from the age at baseline. The reason for quitting smoking was further investigated among former smokers. Alcohol consumption was inquired about frequency and volume, while food consumption was collected on frequency. Physical activity levels were calculated by multiplying the metabolic equivalent tasks (METs) values by the duration of each activity, and then summing them [15]. Pre-bronchodilator lung function was assessed by calibrated spirometers, with two successful blows recorded. Larger FEV1 and FVC values were utilized in our definition of screened COPD (FEV1/FVC < 70%) [16].

### Ascertainment of outcome

All CKB participants were followed up by periodic links through the national inpatient health insurance database, local disease surveillance system, death registry, and active contact with local communities or relatives [13]. In the present study, COPD events were defined as the 10th revision of the International Classification of Diseases: J41-J44 [17, 18]. All participants were followed until death, loss to follow-up, or global censoring date of December 31, 2018.

### Statistical analysis

The weight change for the primary analysis were categorized into six groups: (1) < −5.0, (2) −5.0 ~ −0.1, (3) 0 ~ 4.9, (4) 5.0 ~ 9.9, (5) 10.0 ~ 14.9, and (6) ≥ 15.0 kg. Owing to the limited sample size in certain groups, we grouped the population into four groups: (1) < −0.1, (2) 0 ~ 9.9, (3) 10.0 ~ 14.9, and (4) ≥ 15.0 kg in the subsequent joint and subgroup analyses, respectively. For early adulthood BMI, participants were categorized into four groups according to the criteria specific to the Chinese: (1) < 18.5, (2) 18.5 ~ 23.9, (3) 24.0 ~ 27.9, (4) ≥ 28.0 kg/m^2^ [19]. All analyses were performed by sex separately.

Baseline characteristics were compared across these groups using linear or logistic regression models for continuous or categorical variables correspondingly. Linear trends of exposure with respect to baseline characteristics were further assessed by substituting original exposure categories with medians of corresponding categories for participants in models. Distributions of weight change since early adulthood were presented according to early adulthood BMI, and average weight changes across early adulthood BMI groups were adjusted for age and study region.

Cox proportional hazard regression models were employed to estimate hazard ratios (HRs) and 95% confidence intervals (CIs) for the risk of COPD associated with weight change since early adulthood or early adulthood BMI. Cox models were stratified jointly by age groups in 5-year intervals and 10 study areas, with age as the underlying time scale. Model 1 was adjusted for educational attainment and marital status. Model 2 was additionally adjusted for early adulthood BMI or weight change since early adulthood, where appropriate. Four lifestyle factors (smoking, alcohol consumption, physical activity, consumption frequency of meat, fresh vegetables, and fresh fruit) were further included in Model 3, reflecting effects independent of lifestyle factors. No violations of the proportional hazard assumption for weight change since early adulthood or early adulthood BMI were identified. Linear trends of associations were examined using the procedures described above. The relationship between weight change or early adulthood BMI and COPD was further modeled using restricted cubic splines with knots at the 5th, 35th, 65th, and 95th percentiles of the exposure of our interest based on Model 3. Likelihood ratio tests comparing models with and without cubic spline terms were utilized to determine potential non-linearity. The joint effects of weight change and early adulthood BMI were evaluated by deriving the 4-by-4 composite exposure, and the interactions between them were examined using likelihood ratio tests by comparing models with and without their product terms.

We further delved into the associations stratified by sociodemographic (age groups, education level) and lifestyle factors (smoking, physical activity level) at baseline. Among females, the associations were additionally examined stratified by menopause status. The interactions between early adulthood BMI or weight change and the stratifying variables were inspected using likelihood ratio tests and statistical significance was Bonferroni-corrected for multiple comparisons (*P*_interation_ < 0.05/30).

We performed sensitivity analyses by bootstrap our samples 100 times with replacement, further adjusting for household income and occupation, further adjusting for passive smoking and solid fuel use in cooking and heating; excluding participants whose self-reported weight changes were ≥ 2.5 kg within the last year at baseline; excluding participants with follow-up of < 2 years; using a Fine and Gray model, which extends the Cox model to competing risks of non-COPD death; applying the lower limit of normal (LLN) criterion to define screened COPD cases at baseline [20]; and using the World Health Organization (WHO) criterion for grading early adulthood BMI [21].

We used Stata (version 17.0) for statistical analyses. Statistical significance was defined as the two-sided *P*-values < 0.05.

## Results

Among the 332,923 participants, the mean (SD) baseline age was 49.6 (8.9) years, 58.3% were women, and 46.4% were from urban areas. For males, the mean (SD) BMI at age 25 years and baseline was 21.8 (2.3) kg/m^2^ and 23.6 (3.2) kg/m^2^, respectively, and the mean (SD) weight change over the period was 5.1 (9.2) kg. The counterparts for females were 21.8 (2.6) kg/m^2^, 23.9 (3.4) kg/m^2^, and 5.0 (8.3) kg, respectively. Participants who gained more weight tended to live in urban and northern areas, be married, be physically inactive, consume meat and fresh fruit more frequently, and have a higher baseline BMI and a lower early adulthood BMI for both males and females. While males with greater weight gain were younger, with higher education, and less likely to smoke tobacco, females with 0 ~ 9.9 kg weight gain were the youngest, most highly educated, and least likely to smoke (Table 1). Early adulthood BMI was positively associated with baseline BMI and negatively associated with weight change since age 25 (Table S1 & Figure S1).

**Table 1:** Baseline characteristics of participants according to weight change since early adulthood

Characteristics	Weight change since early adulthood (kg)						*P*_trend_
	< −5.0	−5.0 ~ −0.1	0 ~ 4.9	5.0 ~ 9.9	10.0 ~ 14.9	≥ 15.0	
Males							
No. of participants	17,874	24,652	29,651	26,577	20,166	19,844	
Age at baseline, years	54.5	51.0	49.4	49.2	49.0	49.1	< 0.001
Urban area, %	26.1	31.8	39.8	49.4	56.5	65.1	< 0.001
South area, %	62.6	66.1	62.2	59.6	56.3	50.7	< 0.001
Married, %	92.8	94.0	94.5	95.5	96.0	96.2	< 0.001
> 6 years of education, %	58.7	59.6	62.7	65.1	67.0	68.2	< 0.001
Regular smoker^a^, %	79.3	73.4	68.7	64.7	63.2	62.4	< 0.001
Excessive alcohol drinking^b^, %	26.1	24.2	24.6	23.7	23.8	24.3	< 0.001
Physical activity, MET h/d	25.6	25.7	24.7	23.7	22.7	21.4	< 0.001
Food consumption ≥ 4 d/w, %							
Meat	50.1	51.2	53.8	56.6	58.4	60.1	< 0.001
Fresh vegetable	98.7	98.6	98.8	98.9	98.8	98.9	0.004
Fresh fruit	19.4	20.2	22.7	24.7	26.1	27.9	< 0.001
Baseline BMI, kg/m^2^	20.6	21.4	22.6	24.2	25.6	27.7	< 0.001
Early adulthood BMI, kg/m^2^	23.8	22.3	21.8	21.6	21.2	20.6	< 0.001
Females							
No. of participants	19,185	32,308	49,074	44,257	27,570	21,765	
Age at baseline, years	52.7	49.1	47.6	48.4	49.6	50.9	< 0.001
Urban area, %	36.6	40.2	45.9	50.9	54.9	57.9	< 0.001
South area, %	69.9	69.2	64.9	60.4	56.7	48.8	< 0.001
Married, %	90.6	91.4	91.8	92.1	92.5	92.9	< 0.001
> 6 years of education, %	47.6	49.7	51.9	52.5	51.9	50.3	< 0.001
Regular smoker^a^, %	3.3	2.1	1.7	1.7	1.7	2.1	< 0.001
Excessive alcohol drinking^b^, %	1.2	1.0	1.0	1.1	1.2	1.4	< 0.001
Physical activity, MET h/d	22.6	22.1	21.6	21.2	20.8	20.3	< 0.001
Food consumption ≥ 4 d/w, %							
Meat	45.4	45.9	48.0	49.1	49.4	49.8	< 0.001
Fresh vegetable	98.9	99.0	99.1	99.2	99.3	99.1	< 0.001
Fresh fruit	30.7	33.3	35.4	37.1	37.4	37.8	< 0.001
Baseline BMI, kg/m^2^	20.7	21.8	22.9	24.3	25.9	28.4	< 0.001
Early adulthood BMI, kg/m^2^	24.4	22.7	21.8	21.3	20.9	20.3	< 0.001

The distribution of basic characteristics were presented as means or percentages across each category of weight change since early adulthood, with adjustment for age and regions, where appropriate, using either linear regression or logistic regression

^a^Current smokers and former smokers who quit because of illness

^b^Defined as those who drank ≥ 30 g/d of pure alcohol or have stopped drinking

**Fig. 1:**
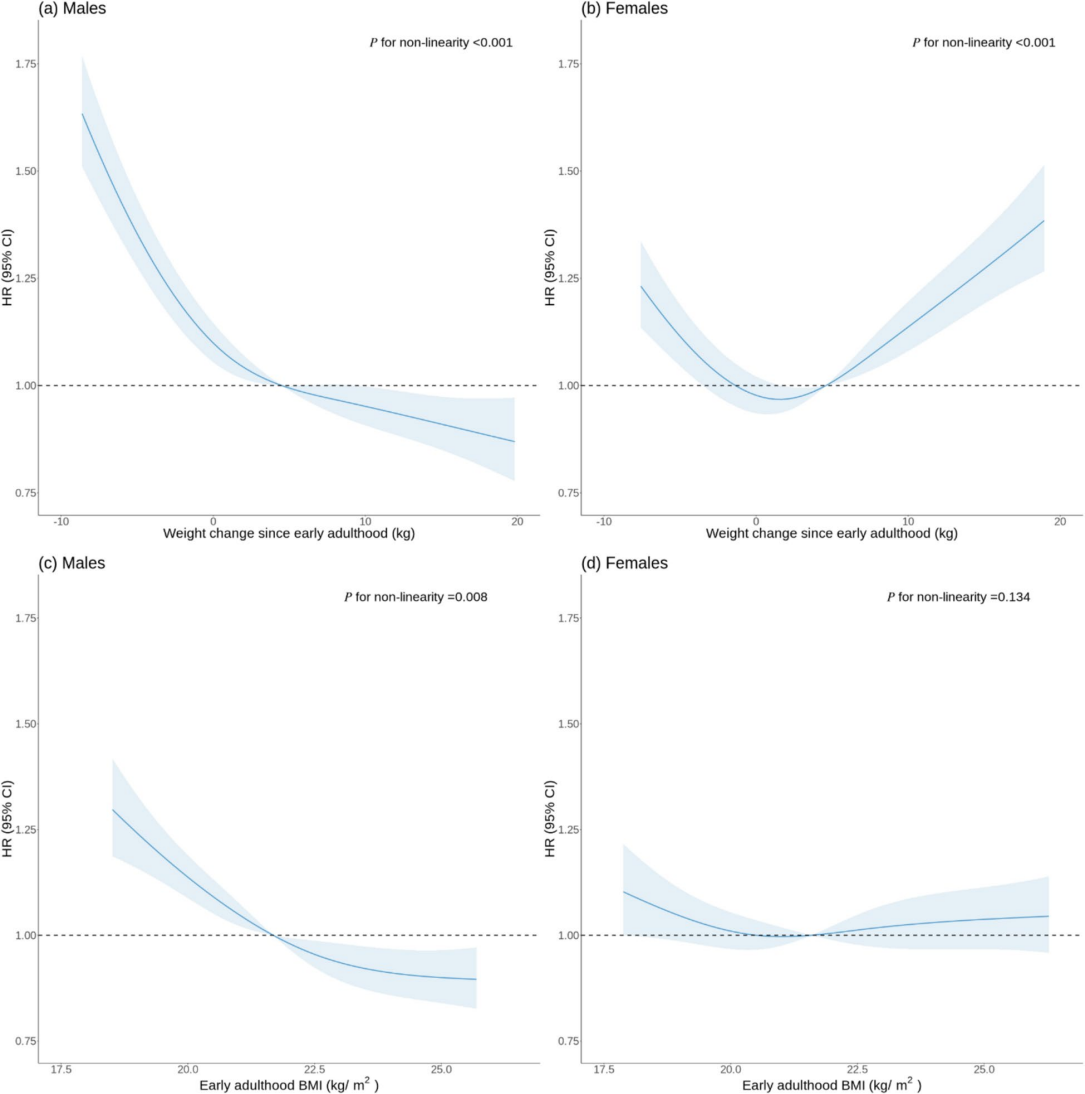
Association of weight change since early adulthood and early adulthood BMI with the risk of COPD: restricted cubic spline (RCS). Note: panel (a-b): association of weight change since early adulthood with the risk of COPD; panel (c-d): association of early adulthood BMI with the risk of COPD; panel (a) & (c): in males; panel (b) & (d): in females. Estimates were adjusted for education, marital status, early adulthood BMI or weight change, and lifestyle factors (smoking, alcohol consumption, physical activity, and dietary habits), stratified by age and region. Former smokers who quitted because of illness were classified as current smokers. Solid lines represent multivariable-adjusted hazard ratios, with the shaded area showing 95% confidence intervals. Reference lines indicating no association were plotted as the dashed lines at a hazard ratio of 1.00. The reference of weight change since early adulthood and early adulthood BMI was set at median values, namely, weight change of 4.5 kg for males and 4.6 kg for females, and early adulthood BMI of 21.7 kg/m2 for males and 21.6 kg/m2 for females. Since estimates around the spline edges were unstable, only graphs of weight change or BMI falling in the 5% to 95% range were presented

Over a mean (minimum–maximum, SD) follow-up of 11.9 (0.0–14.5, 1.9) years, we documented 3,732 and 3,154 incident COPD events in males and females, respectively. Among males, after adjustment for educational attainment, marital status, and early adulthood BMI, the HRs (95% CIs) for participants of < −5.0, −5.0 ~ −0.1, 5.0 ~ 9.9, 10.0 ~ 14.9, and ≥ 15.0 kg changes in weight were 1.59 (1.44, 1.76), 1.12 (1.01, 1.24), 0.85 (0.76, 0.96), 0.93 (0.82, 1.06) and 0.81 (0.70, 0.93), respectively, compared with those with 0 ~ 4.9 kg weight gain. The estimates remained robust after further adjustment for lifestyle factors. The fully-adjusted HRs (95% CIs) for females were 1.33 (1.18, 1.50), 1.04 (0.93, 1.17), 1.11 (0.99, 1.25), 1.24 (1.09, 1.41), and 1.44 (1.26, 1.65), respectively (Table 2). In males, we observed a monotonically decreasing relationship between weight change and COPD risk. In contrast, in females, a U-shaped association was identified (both *P* for non-linearity < 0.001) (Fig. 1).

**Table 2:** Association between weight change since early adulthood and risk of COPD

Weight change (kg)	< −5.0	−5.0 ~ −0.1	0 ~ 4.9	5.0 ~ 9.9	10.0 ~ 14.9	≥ 15.0	*P*_*trend*_
Males							
No. of case	1,086	814	711	478	356	287	
Incidence density (/1000 person-year)	5.37	2.83	2.03	1.52	1.48	1.22	
Model 1	1.44 (1.31, 1.59)	1.09 (0.98, 1.21)	1.00	0.87 (0.77, 0.97)	0.96 (0.85, 1.09)	0.86 (0.75, 0.99)	< 0.001
Model 2	1.59 (1.44, 1.76)	1.12 (1.01, 1.24)	1.00	0.85 (0.76, 0.96)	0.93 (0.82, 1.06)	0.81 (0.70, 0.93)	< 0.001
Model 3	1.50 (1.36, 1.67)	1.09 (0.99, 1.21)	1.00	0.87 (0.77, 0.97)	0.95 (0.83, 1.08)	0.83 (0.72, 0.96)	< 0.001
Females							
No. of case	596	535	606	598	429	390	
Incidence density (/1000 person-year)	2.62	1.37	1.02	1.12	1.29	1.50	
Model 1	1.36 (1.21, 1.52)	1.05 (0.94, 1.18)	1.00	1.12 (1.00, 1.25)	1.25 (1.10, 1.42)	1.47 (1.29, 1.67)	0.052
Model 2	1.40 (1.24, 1.58)	1.06 (0.95, 1.20)	1.00	1.11 (0.99, 1.24)	1.23 (1.09, 1.40)	1.43 (1.25, 1.63)	0.146
Model 3	1.33 (1.18, 1.50)	1.04 (0.93, 1.17)	1.00	1.11 (0.99, 1.25)	1.24 (1.09, 1.41)	1.44 (1.26, 1.65)	0.021

Data were presented as hazard ratios (95% CIs) unless otherwise stated. In Model 1, hazard ratios were adjusted for education and marital status; Model 2 was further adjusted for early adulthood BMI based on Model 1; In Model 3, four lifestyle factors (smoking, alcohol consumption, physical activity, and dietary habits) were further included. Former smokers who quitted because of illness were classified as current smokers. The analyses were stratified according to age and region. The *P* value for sex interaction was < 0.001 in Model 3

Among males, compared with those with an early adulthood BMI of 18.5 ~ 23.9 kg/m^2^, after full adjustment for potential confounders, especially for weight change, the HRs (95% CIs) for participants in the < 18.5, 24.0 ~ 27.9, and ≥ 28.0 kg/m^2^ early adulthood BMI groups were 1.32 (1.15, 1.53), 0.86 (0.78, 0.94), and 0.95 (0.73, 1.23), respectively. The corresponding HRs (95% CIs) for females were 1.13 (1.00, 1.28), 1.01 (0.92, 1.11), and 1.17 (0.96, 1.42), respectively (Table S2). In males, the curve for early adulthood BMI and COPD risk trended downward and leveled off at a BMI of 24.0 kg/m^2^ (*P* for non-linearity = 0.008). In contrast, early adulthood BMI exhibited a steady linear association with COPD risk in females (*P* for non-linearity = 0.134) (Fig. 1).

Interactions between early adulthood BMI and weight change on COPD risk were observed for females (*P*_interaction_ = 0.029). The effect of weight loss on COPD was more substantial in the < 18.5 kg/m^2^ early adulthood BMI group, while the risk of COPD associated with weight gain was higher in the ≥ 24.0 kg/m^2^ early adulthood BMI group. Similar findings were observed among males, although interactions were not statistically significant (*P*_interaction_ = 0.515). Besides, weight gain in the < 18.5 kg/m^2^ early adulthood BMI group and weight loss in the ≥ 28.0 kg/m^2^ early adulthood BMI group were positively associated with COPD risk, compared with a weight gain of 0 ~ 4.9 kg in the 18.5 ~ 23.9 kg/m^2^ early adulthood BMI group (Table 3).

**Table 3:** Joint association of early adulthood BMI and weight change with COPD risk

Early adulthood BMI (kg/m^2^)	Weight change (kg)				*P*_interaction_
	≤ −0.1	0 ~ 4.9	5.0 ~ 9.9	≥ 10.0	
Males					
< 18.5	1.75 (1.24, 2.46)	1.31 (0.97, 1.77)	1.00 (0.70, 1.41)	1.07 (0.87, 1.33)	0.515
18.5 ~ 23.9	1.23 (1.11, 1.36)	1.00	0.86 (0.76, 0.98)	0.89 (0.79, 1.01)	
24.0 ~ 27.9	1.10 (0.98, 1.24)	0.80 (0.62, 1.02)	0.82 (0.59, 1.13)	1.08 (0.79, 1.50)	
≥ 28.0	1.31 (0.99, 1.72)	1.00 (0.32, 3.12)	1.42 (0.35, 5.72)	2.92 (0.94, 9.11)	
Females					
< 18.5	1.56 (1.04, 2.34)	1.43 (1.07, 1.90)	1.50 (1.20, 1.89)	1.28 (1.07, 1.54)	0.029
18.5 ~ 23.9	1.21 (1.07, 1.38)	1.00	1.10 (0.96, 1.25)	1.41 (1.24, 1.60)	
24.0 ~ 27.9	1.13 (0.99, 1.30)	1.01 (0.82, 1.25)	1.26 (0.99, 1.60)	1.60 (1.25, 2.04)	
≥ 28.0	1.27 (1.02, 1.59)	1.45 (0.83, 2.52)	0.63 (0.16, 2.52)	2.30 (0.95, 5.57)	

Hazard ratios were estimated by deriving the 4-by-4 composite exposure of early adulthood BMI and weight change. Results were based on Model 3, adjusted for education, marital status, and lifestyle factors (smoking, alcohol consumption, physical activity, and dietary habits), stratified by age and region. Former smokers who quitted because of illness were classified as current smokers

We also analyzed the associations between weight change since early adulthood or early adulthood BMI and total COPD incidence according to other potential baseline risk factors. The associations were considered consistent across subgroups stratified by age, education, smoking status, and level of physical activity (all *P*_interaction_ > 0.05). Among male non-current smokers, a BMI ≥ 28.0 kg/m^2^ in early adulthood was associated with an increased risk of COPD (HR = 1.76; 95% CI: 1.01, 3.08). We also observed that increased COPD risk caused by obesity was no longer statistically significant among females with higher levels of physical activity (Tables S3 & S4).

In the sensitivity analyses, the associations did not alter appreciably with bootstrap with replacement, further adjusting for household income and occupation, further adjustment for passive smoking and solid fuel use in cooking and heating, excluding participants with < 2 years of follow-up, considering the competing risk of non-COPD mortality, or applying the LLN criterion to define screened COPD cases at baseline. After excluding participants who self-reported a ≥ 2.5 kg weight change within the last year at baseline, the effects of < −5.0 and ≥ 15.0 kg changes in weight and < 18.5 kg/m^2^ early adulthood BMI on COPD became less pronounced overall (Tables S5 & S6). Applying the WHO criterion for BMI grading had a minimal impact on the results, although the risk of COPD conferred by a high BMI at the age of 25 became more evident (Table S7 & S8).

## Discussion

In this large prospective study, we observed an inverse association of COPD risk with adulthood weight change and early adulthood BMI in males. In contrast, a U-shaped association of weight change with COPD risk was identified in females; namely, extreme weight changes, whether loss or gain, were associated with increased COPD risk. Early adulthood underweight was marginally associated with elevated COPD risk in females. There were synergetic effects of early adulthood BMI and weight change on COPD risk in females. The patterns also revealed potential heterogeneity in male non-current smokers and females with higher levels of physical activity regarding early adulthood BMI and COPD risk.

Our study was the first to assess the impact of weight change from early through middle or late adulthood, as well as BMI in early adulthood, on COPD risk. Our findings consistently demonstrated that excessive weight loss and underweight status in early adulthood were associated with an elevated risk of COPD among both males and females. Low weight indicates malnutrition and a paucity of muscular mass and weight decline is invariably accompanied by muscle loss, potentially leading to diminished respiratory muscle strength. Previous research has underscored the critical role of nutrition and extra-thoracic muscle mass in COPD management [22]. A national cross-sectional study of Chinese individuals aged 20 years or older also reported that being underweight was a risk factor for COPD [23]. The study further indicated interventions targeting underweight and weight loss for COPD prevention should be initiated early in life.

Previous studies have shown that being overweight or obese are well-established risk factors for mortality and cardiovascular diseases, even in early adulthood [24–27]. A longitudinal study involving 857 Australian participants demonstrated incident obesity during follow-up was associated with an accelerated decline in post-bronchodilator FEV1 and FVC from age 45–53 years [28]. The study likewise revealed that too much weight gain during adulthood was linked to increased COPD risk in female participants, irrespective of menopausal status. Adipose tissue can be an active endocrine and paracrine organ, which secretes various cytokines, including leptin, adiponectin, interleukin-6, and tumor necrosis factor-α [29]. These bioactive substances can induce multiple physiological effects, such as pro-inflammation, resulting in structural changes and dysfunction in the body [29]. In addition, the accumulation of fat in the chest wall and abdomen tends to restrict the capacity and endurance of gas exchange [30]. However, the finding was not sustained in males. Weight gain and obesity advantages were observed referring to COPD risk, which was consistent with the so-called “obesity paradox” [31]. The underlying mechanism for the “obesity paradox” is still under debate. Some believe that not all fat is equal, with fat type, distribution, and function playing crucial roles [32]. The proportion of muscular mass is also an explanation [8]. Another longitudinal study of 654 young Australian adults found adjustment for childhood lean body mass eliminated any apparent positive influence of childhood BMI on FEV1 or FVC at age 27 ~ 36 years [33]. Furthermore, we found the effect of weight changes on COPD became less prominent after excluding participants who recently experienced relative weight fluctuations. Excess weight change in the short term might incur a higher risk of COPD, which suggests maintaining steady and gradual weight changes is generally more beneficial for pulmonary health.

Overall, the effect of weight loss and gain on COPD was strengthened in the underweight and obese population in early adulthood, respectively, whether their BMI was categorized by Chinese or WHO’s criterion. In a Danish population-based longitudinal study of men aged 25 ~ 48 years, an inverse relationship between current BMI and lung function was seen among those currently overweight and obese, whereas the association vanished in the non-obese [34]. A previous study in smokers followed for a median period of 6 years found the ratio of FEV1/FVC improved with weight gain among those with a BMI of 18.5–30 kg/m^2^, while it was stable among the obese [35]. In addition, excessive weight gain and early obesity were positively associated with COPD risk in the analysis of males. The findings further stressed the importance of sophisticated weight management, suggesting the optimal range of weight change should be determined based on early adulthood weight. To be specific, the underweight should avoid more weight loss, while weight loss is expected in the obese. In addition, the risk of COPD led by underweight and obesity could not be fully offset by later weight catch-up and loss, respectively. Thus, advancing the window of weight management appears necessary from the perspective of COPD prevention.

The stratified analysis revealed most of the associations were unlikely to be confounded. However, among male non-current smokers, there presented positive associations between young obesity and COPD risk, which contrasted with the associations observed in the overall male population. Therefore, we caution against the negative association of young adulthood obesity with COPD, especially for non-current smokers. Furthermore, associations between obesity and COPD risk faded into statistical insignificance among females active in physical activity, suggesting physical activity may attenuate this relationship. Engaging in exercise and increasing muscle mass may help neutralize the harmful effects of obesity. A previous CKB study also observed that being overweight was associated with a lower risk of COPD only among those who were physically active [8].

This study has several strengths worthy of mention. The study was relatively abundant in the sample size, so the joint effect of weight change and BMI in early adulthood could be first inspected simultaneously. All analyses could be conducted in males and females separately, considering the physiological disparity between the sexes. Also, subgroup analyses were enabled among participants according to potential confounders, mainly smoking status. Prospective design with long-term follow-up and outcome linkage to extensive datasets with wide coverage were also highlights of our study. Sorts of sensitivity analyses enhanced the robustness of our primary findings.

The limitations of note are as follows. The sensitivity analysis excluding recent weight change suggests patterns of weight change may matter. However, our study only included weight data from two timepoints and the findings only reflected total weight change over years, rather than patterns of change. Like in most previous observational studies, weight in early adulthood was collected based on recall in our study, which may introduce information bias [2, 36, 37]. However, the baseline self-reported values showed good consistency with those obtained at the first resurvey with all Pearson's correlation coefficient of > 0.75 across weight fluctuation since early adulthood groups and baseline age groups (data not shown) among participants attending both surveys [27]. Furthermore, participants who can recall their early weight may be younger and have better social-economic conditions, thus our results should be extrapolated cautiously. Routine spirometry was not feasible within such a large cohort, necessitating reliance on existing database linkages. Although the CKB study has ascertained outcomes through linkages to health datasets with nearly full coverage, underdiagnosis of COPD is common in China [38], especially for the mild subtypes. In addition, comorbidities among the obese may mask breathing problems [39], leading to results that tend towards null and even reverse directions. Some misclassification of incident COPD remains possible, though we adjudicated ~ 1,000 randomly selected cases in 2016 by reviewing hospital medical records and 85% were supported [40]. And reverse causation, like weight loss caused by preclinical COPD, may have contributed to the above findings. Nonetheless, the analyses were repeated excluding participants with follow-up of < 2 years, and the primary results proved reliable. We additionally adjusted for occupational category, but residual confounding due to unmeasured workplace exposures cannot be fully excluded. Although we controlled for passive smoking and solid fuel use in sensitivity analysis, the likelihood of residual confounding due to uncontrolled environmental variables, such as outdoor air pollution, still existed. Notwithstanding, a previous CKB study observed that personal exposure levels of fine particles correlated more strongly with household levels than community levels [41]. Lifestyle factors were controlled for in our study, which demonstrated the robustness of our findings but was accompanied by the possibility of underestimation. The CKB study areas were selected to represent major regional differences in disease patterns, lifestyle, and economic levels. Therefore, our findings are likely generalizable to diverse populations across China. Whether the conclusion can be generalized to other populations remains to be validated.

## Conclusion

Weight change and early adulthood BMI were inversely linked to COPD risk in males, while females faced an elevated risk of COPD with either extreme weight loss or weight gain. Our study highlights the importance of maintaining a healthy weight beginning in young adulthood, with a specific emphasis on promoting weight gain in the underweight and weight loss in the obese as potential measures for reducing COPD risk. This further underscores that weight fluctuations should be maintained steadily and softly.

## Supplementary Information

Supplementary Material 1.

## Data Availability

The CKB study is a global resource for the investigation of lifestyle, environmental, blood biochemical, and genetic factors as determinants of common diseases. The CKB study group is committed to making the cohort data available to the scientific community in China, UK, and worldwide to advance knowledge about the causes, prevention, and treatment of disease. For detailed information on what data is currently available to open access for users and how to apply for it, visit: [https://www.ckbiobank.org/data-access](https://www.ckbiobank.org/data-access).
