# Supplementary material for: The COPD risk associated with adulthood weight change and early adulthood BMI: a prospective cohort study
Source: BMC Public Health. 2025 Nov 22;26:364. doi: 10.1186/s12889-025-25582-z (PMC12849291; doi:10.1186/s12889-025-25582-z)
Supplement: Supplementary file 1 — Supplementary Material 1. [file 12889_2025_25582_MOESM1_ESM.docx]

**Supplementary Materials**

Supplementary material for the manuscript entitled “The COPD risk associated with adulthood weight change and early adulthood BMI: a prospective cohort study”

Catalogue

[Supplementary Materials 1](#_Toc209346059)

[Members of the China Kadoorie Biobank collaborative group 2](#_Toc209346060)

[Table S1. Baseline characteristics of participants according to early adulthood BMI. 3](#_Toc209346061)

[Figure S1. Distribution of weight change since early adulthood according to early adulthood BMI. 4](#_Toc209346062)

[Table S2. Association between early adulthood BMI and risk of COPD. 5](#_Toc209346063)

[Table S3. Subgroup analysis for weight change since early adulthood and COPD risk. 6](#_Toc209346064)

[Table S4. Subgroup analysis for early adulthood BMI and COPD risk. 8](#_Toc209346065)

[Table S5. Sensitivity analysis for the association between weight change since early adulthood and COPD. 10](#_Toc209346066)

[Table S6. Sensitivity analysis for the association between early adulthood BMI and COPD. 12](#_Toc209346067)

[Table S7. Sensitivity analysis for the association between early adulthood BMI and risk of COPD. 13](#_Toc209346068)

[Table S8. Sensitivity analysis for the joint association of early adulthood BMI and weight change with COPD risk. 14](#_Toc209346069)

**Members of the China Kadoorie Biobank collaborative group**

**International Steering Committee:** Junshi Chen, Zhengming Chen (PI), Robert Clarke, Rory Collins, Liming Li (PI), Jun Lv, Richard Peto, Robin Walters.

**International Co-ordinating Centre, Oxford:** Daniel Avery, Maxim Barnard, Derrick Bennett, Ruth Boxall, Yiping Chen, Zhengming Chen, Jonathan Clarke; Robert Clarke, Huaidong Du, Ahmed Edris Mohamed, Hannah Fry, Yani Huang, Pek Kei Im, Andri Iona, Christiana Kartsonaki, Kshitij Kolhe, Hubert Lam, Kuang Lin, James Liu, Iona Millwood, Sam Morris, Qunhua Nie, Alfred Pozarickij, Maryam Rahmati, Paul Ryder, Maruf Sarder, Dan Schmidt, Becky Stevens, Robin Walters, Baihan Wang, Lin Wang, Neil Wright, Ling Yang, Xiaoming Yang, Pang Yao.

**National Co-ordinating Centre, Beijing:** Xiao Han, Can Hou, Qingmei Xia, Chao Liu, Jun Lv, Pei Pei, Dianjianyi Sun, Canqing Yu, Lang Pan.

**10 Regional Co-ordinating Centres:**

**Qingdao CDC:** Zengchang Pang, Ruqin Gao, Shanpeng Li, Haiping Duan, Shaojie Wang, Yongmei Liu, Ranran Du, Yajing Zang, Liang Cheng, Xiaocao Tian, Hua Zhang, Yaoming Zhai, Feng Ning, Xiaohui Sun, Feifei Li. **Licang CDC:** Silu Lv, Junzheng Wang, Wei Hou. **Heilongjiang Provincial CDC:** Wei Sun, Shichun Yan, Xiaoming Cui. **Nangang CDC:** Chi Wang, Zhenyuan Wu,Yanjie Li, Quan Kang. **Hainan Provincial CDC:** Huiming Luo, Tingting Ou. **Meilan CDC:** Xiangyang Zheng, Zhendong Guo, Shukuan Wu, Yilei Li, Huimei Li. **Jiangsu Provincial CDC:** Ming Wu, Yonglin Zhou, Jinyi Zhou, Ran Tao, Jie Yang, Jian Su. **Suzhou CDC:** Fang Liu, Jun Zhang, Yihe Hu, Yan Lu, Liangcai Ma, Aiyu Tang, Shuo Zhang, Jianrong Jin, Jingchao Liu. **Guangxi Provincial CDC:** Mei Lin, Zhenzhen Lu. **Liuzhou CDC:** Lifang Zhou, Changping Xie, Jian Lan,Tingping Zhu,Yun Liu, Liuping Wei, Liyuan Zhou, Ningyu Chen, Yulu Qin, Sisi Wang. **Sichuan Provincial CDC:** Xianping Wu, Ningmei Zhang, Xiaofang Chen, Xiaoyu Chang. **Pengzhou CDC:** Mingqiang Yuan, Xia Wu, Xiaofang Chen, Wei Jiang, Jiaqiu Liu, Qiang Sun. **Gansu Provincial CDC:** Faqing Chen, Xiaolan Ren, Caixia Dong. **Maiji CDC:** Hui Zhang, Enke Mao, Xiaoping Wang, Tao Wang, Xi zhang. **Henan Provincial CDC:** Kai Kang, Shixian Feng, Huizi Tian, Lei Fan. **Huixian CDC:** XiaoLin Li, Huarong Sun, Pan He, Xukui Zhang. **Zhejiang Provincial CDC:** Min Yu, Ruying Hu, Hao Wang. **Tongxiang CDC**: Xiaoyi Zhang, Yuan Cao, Kaixu Xie, Lingli Chen, Dun Shen. **Hunan Provincial CDC:** Xiaojun Li, Donghui Jin, Li Yin, Huilin Liu, Zhongxi Fu. **Liuyang CDC:** Xin Xu, Hao Zhang, Jianwei Chen,Yuan Peng, Libo Zhang, Chan Qu.

# Table S1. Baseline characteristics of participants according to early adulthood BMI.

| Characteristics | Early adulthood BMI (kg/m^2^) | | | | *P*_trend_ |
| --- | --- | --- | --- | --- | --- |
|  | <18.5 | 18.5~23.9 | 24.0~27.9 | ≥28.0 |  |
| Males |  |  |  |  |  |
| No. of participants | 7,163 | 110,453 | 19,455 | 1,693 |  |
| Age at baseline, years | 49.2 | 49.9 | 52.3 | 51.3 | <0.001 |
| Urban area, % | 54.4 | 44.4 | 40.9 | 49.5 | <0.001 |
| South area, % | 66.3 | 60.2 | 57.3 | 47.0 | <0.001 |
| Married, % | 93.7 | 95.0 | 94.3 | 93.5 | 0.089 |
| >6 years of education, % | 66.7 | 63.8 | 59.1 | 61.3 | <0.001 |
| Regular smoker^¶^, % | 67.2 | 68.1 | 70.4 | 68.5 | <0.001 |
| Excessive alcohol drinking^※^, % | 23.3 | 24.0 | 26.7 | 28.5 | <0.001 |
| Physical activity, MET h/d | 23.0 | 24.0 | 24.8 | 24.4 | <0.001 |
| Food consumption ≥4 d/w, % |  |  |  |  |  |
| Meat | 55.3 | 55.0 | 54.0 | 54.2 | 0.003 |
| Fresh vegetable | 98.4 | 98.8 | 98.7 | 99.2 | 0.086 |
| Fresh fruit | 23.6 | 23.8 | 22.9 | 24.2 | 0.083 |
| Baseline BMI, kg/m^2^ | 21.9 | 23.4 | 25.1 | 27.3 | <0.001 |
| Weight change since early adulthood, kg | 12.0 | 5.7 | 0.0 | -5.8 | <0.001 |
| Females |  |  |  |  |  |
| No. of participants | 18,042 | 138,065 | 34,886 | 3,166 |  |
| Age at baseline, years | 48.5 | 48.7 | 51.0 | 53.8 | <0.001 |
| Urban area, % | 55.4 | 48.1 | 43.7 | 39.1 | <0.001 |
| South area, % | 66.0 | 62.4 | 59.3 | 58.8 | <0.001 |
| Married, % | 91.4 | 92.1 | 91.9 | 89.9 | 0.364 |
| >6 years of education, % | 54.0 | 52.0 | 46.9 | 42.0 | <0.001 |
| Regular smoker^¶^, % | 2.2 | 1.9 | 2.2 | 3.0 | <0.001 |
| Excessive alcohol drinking^※^, % | 1.3 | 1.0 | 1.1 | 1.6 | 0.091 |
| Physical activity, MET h/d | 20.8 | 21.4 | 22.0 | 22.0 | <0.001 |
| Food consumption ≥4 d/w, % |  |  |  |  |  |
| Meat | 48.9 | 48.2 | 46.8 | 46.2 | <0.001 |
| Fresh vegetable | 99.0 | 99.1 | 99.1 | 98.6 | 0.395 |
| Fresh fruit | 37.1 | 36.0 | 33.1 | 33.9 | <0.001 |
| Baseline BMI, kg/m^2^ | 22.0 | 23.6 | 25.5 | 27.0 | <0.001 |
| Weight change since early adulthood, kg | 10.8 | 5.7 | 0.5 | -5.3 | <0.001 |
| Note: The distribution of basic characteristics was presented as means or percentages across each category of weight change since early adulthood, with adjustment for age and regions, where appropriate, using either linear regression or logistic regression.  ^¶^ Current smokers and former smokers who quit because of illness.  ^※^ Defined as those who drank ≥30 g/d of pure alcohol or have stopped drinking. | | | | | |


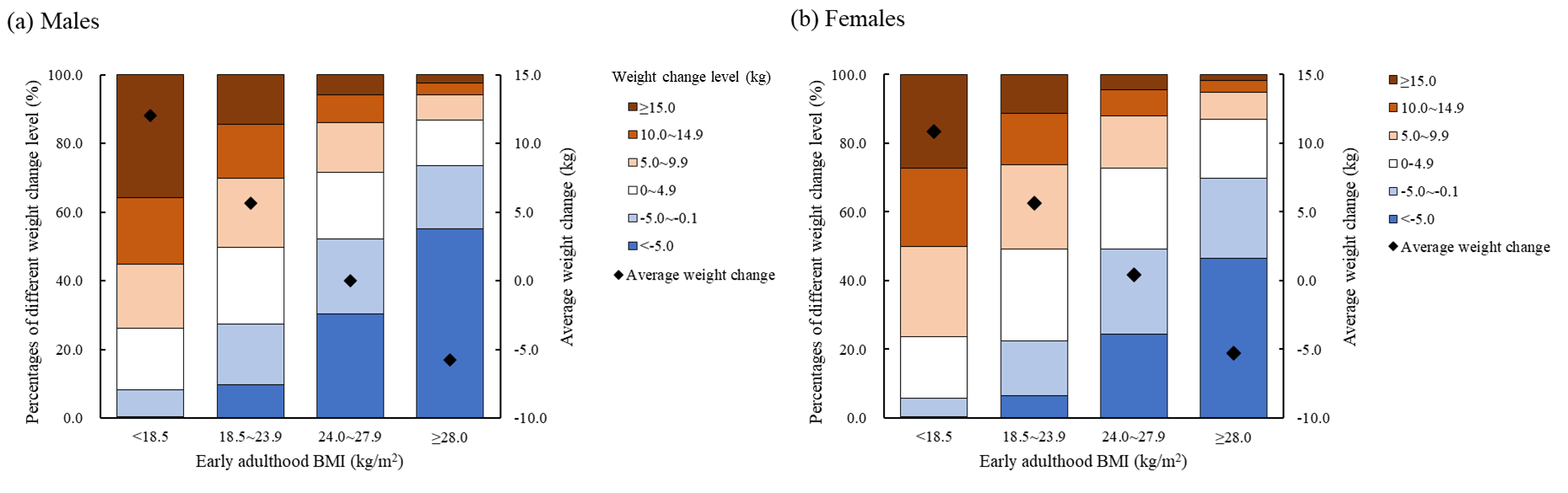


# Figure S1. Distribution of weight change since early adulthood according to early adulthood BMI.

Note: Distributions of weight change since early adulthood were presented according to early adulthood BMI, and average weight changes across early adulthood BMI groups were presented as diamonds, adjusted for age and region.

# Table S2. Association between early adulthood BMI and risk of COPD.

| Early adulthood BMI (kg/m^2^) | <18.5 | 18.5~23.9 | 24.0~27.9 | ≥28.0 | *P_trend_* |
| --- | --- | --- | --- | --- | --- |
| Males |  |  |  |  |  |
| No. of case | 213 | 2,789 | 666 | 64 |  |
| Incidence density (/1000 person-year) | 2.55 | 2.14 | 2.95 | 3.32 |  |
| Model 1 | 1.12 (0.97, 1.28) | 1.00 | 1.00 (0.92, 1.09) | 1.29 (1.00, 1.65) | 0.889 |
| Model 2 | 1.33 (1.16, 1.54) | 1.00 | 0.85 (0.78, 0.93) | 0.93 (0.72, 1.20) | <0.001 |
| Model 3 | 1.32 (1.15, 1.53) | 1.00 | 0.86 (0.78, 0.94) | 0.95 (0.73, 1.23) | <0.001 |
| Females |  |  |  |  |  |
| No. of case | 333 | 2,019 | 684 | 118 |  |
| Incidence density (/1000 person-year) | 1.54 | 1.21 | 1.63 | 3.19 |  |
| Model 1 | 1.17 (1.04, 1.32) | 1.00 | 0.99 (0.91, 1.08) | 1.14 (0.95, 1.38) | 0.324 |
| Model 2 | 1.15 (1.02, 1.30) | 1.00 | 1.01 (0.92, 1.10) | 1.18 (0.97, 1.44) | 0.758 |
| Model 3 | 1.13 (1.00, 1.28) | 1.00 | 1.01 (0.92, 1.11) | 1.17 (0.96, 1.42) | 0.900 |
| Note: Data were presented as hazard ratios (95% CIs) unless otherwise stated. In Model 1, hazard ratios were adjusted for education and marital status; Model 2 were further adjusted for weight change based on Model 1; In Model 3, four lifestyle factors (smoking, alcohol consumption, physical activity, and dietary habits) were further included. Former smokers who quitted because of illness were classified as current amokers. The analyses were stratified according to age and region. The *P* value for sex interaction was 0.711 in Model 3. | | | | | |

# Table S3. Subgroup analysis for weight change since early adulthood and COPD risk.

| Subgroups | Weight change (kg) | | | | *P*_interaction_ | |
| --- | --- | --- | --- | --- | --- | --- |
|  | ≤-0.1 | 0~4.9 | 5.0~9.9 | ≥10.0 |  | |
| Males |  |  |  |  |  | |
| Age, years |  |  |  |  | 0.090 | |
| <45 | 0.87 (0.63, 1.20) | 1.00 | 0.66 (0.45, 0.96) | 0.74 (0.53, 1.04) |  | |
| 45~60 | 1.29 (1.13, 1.47) | 1.00 | 0.93 (0.78, 1.09) | 0.85 (0.72, 1.00) |  | |
| ≥60 | 1.32 (1.15, 1.51) | 1.00 | 0.86 (0.72, 1.03) | 1.02 (0.86, 1.21) |  | |
| Education level |  |  |  |  | 0.111 | |
| Primary school and below | 1.26 (1.13, 1.41) | 1.00 | 0.91 (0.79, 1.06) | 1.00 (0.86, 1.16) |  | |
| Middle school and above | 1.28 (1.10, 1.49) | 1.00 | 0.81 (0.67, 0.97) | 0.81 (0.68, 0.96) |  | |
| Tobacco smoking |  |  |  |  | 0.121 | |
| Non-current smoker | 1.42 (1.11, 1.80) | 1.00 | 0.86 (0.64, 1.16) | 1.01 (0.77, 1.33) |  | |
| Quitter | 1.49 (1.13, 1.96) | 1.00 | 0.89 (0.64, 1.23) | 0.88 (0.65, 1.19) |  | |
| Reason for quitting |  |  |  |  | 0.805 | |
| Physical illness | 1.57 (1.14, 2.15) | 1.00 | 1.05 (0.72, 1.53) | 1.04 (0.73, 1.49) |  | |
| Other reasons | 1.22 (0.85, 1.76) | 1.00 | 0.71 (0.46, 1.11) | 0.76 (0.51, 1.14) |  | |
| Current smoker | 1.21 (1.09, 1.34) | 1.00 | 0.87 (0.76, 1.00) | 0.91 (0.80, 1.04) |  | |
| No. of cigarettes/d |  |  |  |  | 0.725 | |
| ≤10 | 1.16 (0.93, 1.44) | 1.00 | 0.80 (0.59, 1.06) | 0.82 (0.62, 1.08) |  | |
| 11-20 | 1.52 (1.15, 2.02) | 1.00 | 1.14 (0.79, 1.65) | 1.08 (0.72, 1.60) |  | |
| ≥ 10 | 1.17 (1.03, 1.33) | 1.00 | 0.86 (0.72, 1.02) | 0.92 (0.78, 1.09) |  | |
| Years of smoking history |  |  |  |  | 0.527 | |
| < 20 | 1.11 (0.70, 1.76) | 1.00 | 0.82 (0.47, 1.43) | 0.81 (0.48, 1.37) |  | |
| 20-35 | 1.28 (1.08, 1.51) | 1.00 | 0.91 (0.73, 1.13) | 0.87 (0.70, 1.08) |  | |
| ≥ 35 | 1.16 (1.01, 1.34) | 1.00 | 0.85 (0.70, 1.02) | 0.96 (0.80, 1.15) |  | |
| Physical activity ^¶^ |  |  |  |  | 0.855 | |
| Low | 1.27 (1.12, 1.42) | 1.00 | 0.89 (0.76, 1.03) | 0.90 (0.78, 1.03) |  | |
| High | 1.26 (1.09, 1.44) | 1.00 | 0.84 (0.69, 1.00) | 0.94 (0.79, 1.12) |  | |
| Females |  |  |  |  |  | |
| Age, years |  |  |  |  | 0.502 | |
| <45 | 1.16 (0.88, 1.53) | 1.00 | 1.03 (0.78, 1.36) | 1.29 (0.97, 1.72) |  | |
| 45~60 | 1.15 (0.99, 1.34) | 1.00 | 1.13 (0.96, 1.33) | 1.46 (1.25, 1.69) |  | |
| ≥60 | 1.18 (1.00, 1.40) | 1.00 | 1.15 (0.94, 1.40) | 1.21 (1.00, 1.46) |  | |
| Education level |  |  |  |  | 0.958 | |
| Primary school and below | 1.14 (1.01, 1.28) | 1.00 | 1.11 (0.97, 1.27) | 1.31 (1.15, 1.49) |  | |
| Middle school and above | 1.23 (0.99, 1.51) | 1.00 | 1.13 (0.91, 1.40) | 1.40 (1.14, 1.72) |  | |
| Tobacco smoking |  |  |  |  | 0.693 | |
| Non-current smoker | 1.17 (1.05, 1.31) | 1.00 | 1.12 (0.99, 1.26) | 1.37 (1.22, 1.54) |  | |
| Quitter | 0.73 (0.37, 1.44) | 1.00 | 0.94 (0.47, 1.86) | 1.34 (0.73, 2.45) |  | |
| Reason for quitting |  |  |  |  | 0.051 | |
| Physical illness | 0.96 (0.44, 2.11) | 1.00 | 1.18 (0.53, 2.61) | 1.57 (0.76, 3.22) |  | |
| Other reasons | 0.36 (0.13, 0.99) | 1.00 | 0.83 (0.32, 2.18) | 0.63 (0.24, 1.66) |  | |
| Current smoker | 1.15 (0.82, 1.60) | 1.00 | 1.23 (0.83, 1.81) | 1.09 (0.73, 1.62) |  | |
| No. of cigarettes/d |  |  |  |  | 0.411 | |
| ≤10 | 1.41 (0.91, 2.17) | 1.00 | 1.24 (0.75, 2.08) | 1.26 (0.75, 2.11) |  | |
| 11-20 | 0.95 (0.41, 2.18) | 1.00 | 1.37 (0.50, 3.80) | 1.70 (0.64, 4.58) |  | |
| ≥ 10 | 0.94 (0.38, 2.30) | 1.00 | 1.35 (0.50, 3.65) | 0.68 (0.24, 1.91) |  | |
| Years of smoking history |  |  |  |  | 0.063 | |
| < 20 | 1.06 (0.39, 2.85) | 1.00 | 1.47 (0.49, 4.37) | 0.95 (0.30, 2.96) |  | |
| 20-35 | 1.32 (0.72, 2.43) | 1.00 | 1.20 (0.59, 2.45) | 0.70 (0.32, 1.54) |  | |
| ≥ 35 | 1.02 (0.63, 1.65) | 1.00 | 1.25 (0.72, 2.18) | 1.32 (0.76, 2.28) |  | |
| Physical activity ^¶^ |  |  |  |  | 0.295 | |
| Low | 1.22 (1.05, 1.42) | 1.00 | 1.12 (0.96, 1.32) | 1.25 (1.07, 1.46) |  | |
| High | 1.11 (0.97, 1.28) | 1.00 | 1.10 (0.94, 1.30) | 1.45 (1.25, 1.70) |  | |
| Menopause |  |  |  |  | 0.828 | |
| No | 1.09 (0.88, 1.35) | 1.00 | 1.06 (0.85, 1.31) | 1.27 (1.03, 1.58) |  | |
| Yes | 1.18 (1.05, 1.33) | 1.00 | 1.14 (1.00, 1.31) | 1.37 (1.20, 1.55) |  | |
| Note: Results were based on Model 3, adjusted for education, marital status, early adulthood BMI, and lifestyle factors (smoking, alcohol consumption, physical activity, and dietary habits), stratified by age and regions, where appropriate. *P* values for interaction were calculated by likelihood ratio test comparing models with and without a cross-product term. | | | | | |  |

^¶^ Divided by sex-specific medians of metabolic equivalent task hours.

# Table S4. Subgroup analysis for early adulthood BMI and COPD risk.

| Subgroups | Early adulthood BMI (kg/m^2^) | | | | *P*_interaction_ |
| --- | --- | --- | --- | --- | --- |
|  | <18.5 | 18.5~23.9 | 24.0~27.9 | ≥28.0 |  |
| Males |  |  |  |  |  |
| Age, years |  |  |  |  | 0.608 |
| <45 | 1.29 (0.80, 2.09) | 1.00 | 1.03 (0.70, 1.51) | 1.50 (0.55, 4.10) |  |
| 45~60 | 1.20 (0.96, 1.51) | 1.00 | 0.80 (0.69, 0.92) | 0.72 (0.44, 1.15) |  |
| ≥60 | 1.44 (1.17, 1.76) | 1.00 | 0.89 (0.79, 1.01) | 1.06 (0.77, 1.46) |  |
| Education level |  |  |  |  | 0.311 |
| Primary school and below | 1.18 (0.97, 1.44) | 1.00 | 0.86 (0.77, 0.95) | 1.03 (0.76, 1.38) |  |
| Middle school and above | 1.54 (1.25, 1.90) | 1.00 | 0.87 (0.74, 1.03) | 0.74 (0.45, 1.23) |  |
| Tobacco smoking |  |  |  |  | 0.138 |
| Non-current smoker | 1.03 (0.70, 1.49) | 1.00 | 0.97 (0.77, 1.24) | 1.76 (1.01, 3.08) |  |
| Quitter | 1.50 (0.98, 2.29) | 1.00 | 0.87 (0.66, 1.14) | 0.54 (0.22, 1.32) |  |
| Reason for quitting |  |  |  |  | 0.627 |
| Physical illness | 1.50 (0.89, 2.51) | 1.00 | 1.01 (0.75, 1.37) | 1.04 (0.47, 2.29) |  |
| Other Reasons | 1.62 (0.95, 2.76) | 1.00 | 0.91 (0.63, 1.33) | 0.55 (0.13, 2.26) |  |
| Current smoker | 1.38 (1.17, 1.63) | 1.00 | 0.84 (0.76, 0.93) | 0.89 (0.66, 1.21) |  |
| No. of cigarettes/d |  |  |  |  | 0.479 |
| ≤10 | 1.60 (1.16, 2.21) | 1.00 | 0.81 (0.64, 1.02) | 1.36 (0.76, 2.43) |  |
| 11-20 | 1.58 (1.02, 2.44) | 1.00 | 0.94 (0.73, 1.22) | 0.72 (0.31, 1.68) |  |
| ≥20 | 1.25 (1.00, 1.56) | 1.00 | 0.82 (0.72, 0.94) | 0.80 (0.54, 1.20) |  |
| Years of smoking history |  |  |  |  |  |
| <20^*^ | 2.59 (1.47, 4.57) | 1.00 | 0.81 (0.48, 1.36) | NA | 0.051 |
| 20-35 | 1.16 (0.85, 1.56) | 1.00 | 0.89 (0.75, 1.06) | 0.86 (0.48, 1.54) |  |
| ≥35 | 1.41 (1.14, 1.76) | 1.00 | 0.82 (0.72, 0.94) | 0.97 (0.68, 1.40) |  |
| Physical activity ^¶^ |  |  |  |  | 0.819 |
| Low | 1.34 (1.12, 1.60) | 1.00 | 0.88 (0.78, 0.99) | 0.90 (0.64, 1.26) |  |
| High | 1.29 (1.00, 1.66) | 1.00 | 0.83 (0.72, 0.95) | 1.01 (0.68, 1.50) |  |
| Females |  |  |  |  |  |
| Age, years |  |  |  |  | 0.183 |
| <45 | 1.42 (1.04, 1.93) | 1.00 | 1.11 (0.84, 1.47) | 0.49 (0.12, 1.99) |  |
| 45~60 | 1.08 (0.91, 1.27) | 1.00 | 1.08 (0.95, 1.23) | 1.06 (0.77, 1.48) |  |
| ≥60 | 1.10 (0.89, 1.36) | 1.00 | 0.90 (0.77, 1.05) | 1.21 (0.93, 1.58) |  |
| Education level |  |  |  |  | 0.156 |
| Primary school and below | 1.06 (0.92, 1.23) | 1.00 | 0.99 (0.89, 1.10) | 1.17 (0.95, 1.45) |  |
| Middle school and above | 1.31 (1.06, 1.62) | 1.00 | 1.09 (0.89, 1.32) | 0.95 (0.52, 1.76) |  |
| Tobacco smoking |  |  |  |  | 0.563 |
| Non-current smoker | 1.10 (0.97, 1.26) | 1.00 | 1.00 (0.90, 1.11) | 1.27 (1.01, 1.58) |  |
| Quitter | 1.01 (0.53, 1.93) | 1.00 | 1.11 (0.63, 1.94) | 1.54 (0.53, 4.41) |  |
| Reason for quitting |  |  |  |  | 0.244 |
| Physical illness | 1.48 (0.75, 2.92) | 1.00 | 1.00 (0.52, 1.94) | 1.31 (0.46, 3.79) |  |
| Other Reasons | 0.60 (0.16, 2.31) | 1.00 | 0.75 (0.31, 1.81) | 2.25 (0.48, 10.60) |  |
| Current smoker | 1.42 (0.96, 2.10) | 1.00 | 1.08 (0.83, 1.42) | 0.83 (0.51, 1.33) |  |
| No. of cigarettes/d |  |  |  |  | 0.354 |
| ≤10 | 1.67 (1.02, 2.71) | 1.00 | 1.10 (0.77, 1.56) | 1.11 (0.63, 1.96) |  |
| 11-20 | 1.06 (0.42, 2.71) | 1.00 | 1.19 (0.61, 2.30) | 0.62 (0.16, 2.46) |  |
| ≥20 | 0.81 (0.24, 2.74) | 1.00 | 1.71 (0.85, 3.44) | 0.71 (0.16, 3.13) |  |
| Years of smoking history |  |  |  |  |  |
| <20^*^ | 0.53 (0.14, 2.10) | 1.00 | 0.59 (0.23, 1.51) | NA | 0.066 |
| 20-35 | 3.31 (1.60, 6.82) | 1.00 | 1.46 (0.90, 2.39) | 0.69 (0.24, 2.01) |  |
| ≥35 | 1.31 (0.77, 2.25) | 1.00 | 1.11 (0.76, 1.61) | 1.04 (0.58, 1.86) |  |
| Physical activity ^¶^ |  |  |  |  | 0.353 |
| Low | 1.15 (0.98, 1.36) | 1.00 | 0.97 (0.85, 1.11) | 1.32 (1.00, 1.73) |  |
| High | 1.11 (0.93, 1.32) | 1.00 | 1.05 (0.92, 1.19) | 1.04 (0.78, 1.39) |  |
| Menopause |  |  |  |  | 0.350 |
| No | 1.25 (0.98, 1.59) | 1.00 | 1.09 (0.89, 1.34) | 0.81 (0.38, 1.72) |  |
| Yes | 1.10 (0.96, 1.26) | 1.00 | 0.99 (0.89, 1.10) | 1.18 (0.96, 1.45) |  |

Note: Results were based on Model 3, adjusted for education, marital status, weight change, and lifestyle factors (smoking, alcohol consumption, physical activity, and dietary habits), stratified by age and region, where appropriate. P values for interaction were calculated by likelihood ratio test comparing models with and without a cross-product term.

^¶^ Divided by sex-specific medians of metabolic equivalent task hours.

^*^ For male smokers with <20 years of smoking history, there were no COPD cases among those with a BMI >=28.0 kg/m^2^ in early adulthood.

# Table S5. Sensitivity analysis for the association between weight change since early adulthood and COPD.

| Weight Change (kg) | <-5.0 | -5.0~-0.1 | 0~4.9 | 5.0~9.9 | 10.0~14.9 | ≥15.0 | *P_l_*_inear_ |
| --- | --- | --- | --- | --- | --- | --- | --- |
| Males |  |  |  |  |  |  |  |
| Model 3 | 1.50 (1.36, 1.67) | 1.09 (0.99, 1.21) | 1.00 | 0.87 (0.77, 0.97) | 0.95 (0.83, 1.08) | 0.83 (0.72, 0.96) | <0.001 |
| Bootstrap 100 times with replacement | 1.50 (1.35, 1.67) | 1.09 (0.99, 1.20) | 1.00 | 0.87 (0.77, 0.97) | 0.95 (0.83, 1.09) | 0.83 (0.71, 0.96) | <0.001 |
| Further adjustment for household income and occupation | 1.51 (1.36, 1.67) | 1.09 (0.99, 1.21) | 1.00 | 0.86 (0.77, 0.97) | 0.95 (0.83, 1.08) | 0.82 (0.71, 0.95) | <0.001 |
| Further adjustment for passive smoking and solid fuel use in cooking and heating | 1.50 (1.36, 1.67) | 1.09 (0.99, 1.21) | 1.00 | 0.86 (0.77, 0.97) | 0.95 (0.83, 1.08) | 0.83 (0.72, 0.96) | <0.001 |
| Exclusion of participants whose weight changed≥2.5kg within the last year | 1.43 (1.27, 1.60) | 1.07 (0.96, 1.20) | 1.00 | 0.84 (0.73, 0.95) | 0.92 (0.80, 1.07) | 0.85 (0.73, 1.00) | <0.001 |
| Exclusion of participants with follow-up <2 years | 1.50 (1.35, 1.67) | 1.09 (0.99, 1.21) | 1.00 | 0.87 (0.77, 0.98) | 0.96 (0.85, 1.10) | 0.85 (0.74, 0.98) | <0.001 |
| Further adjustment for competing risk of non-COPD death | 1.48 (1.33, 1.64) | 1.08 (0.97, 1.19) | 1.00 | 0.87 (0.77, 0.97) | 0.95 (0.83, 1.08) | 0.82 (0.71, 0.95) | <0.001 |
| Definition of COPD using LLN criterion at baseline | 1.50 (1.35, 1.66) | 1.11 (1.00, 1.23) | 1.00 | 0.87 (0.78, 0.98) | 0.95 (0.83, 1.08) | 0.84 (0.72, 0.96) | <0.001 |
| Females |  |  |  |  |  |  |  |
| Model 3 | 1.33 (1.18, 1.50) | 1.04 (0.93, 1.17) | 1.00 | 1.11 (0.99, 1.25) | 1.24 (1.09, 1.41) | 1.44 (1.26, 1.65) | 0.021 |
| Bootstrap 100 times with replacement | 1.33 (1.19, 1.48) | 1.04 (0.92, 1.18) | 1.00 | 1.11 (0.99, 1.25) | 1.24 (1.10, 1.39) | 1.44 (1.26, 1.65) | 0.026 |
| Further adjustment for household income and occupation | 1.33 (1.18, 1.51) | 1.04 (0.93, 1.17) | 1.00 | 1.11 (0.99, 1.25) | 1.24 (1.09, 1.40) | 1.44 (1.26, 1.65) | 0.026 |
| Further adjustment for passive smoking and solid fuel use in cooking and heating | 1.33 (1.18, 1.50) | 1.04 (0.93, 1.17) | 1.00 | 1.11 (0.99, 1.25) | 1.24 (1.09, 1.41) | 1.45 (1.27, 1.65) | 0.019 |
| Exclusion of participants whose weight changed≥2.5kg within the last years | 1.27 (1.10, 1.46) | 1.03 (0.90, 1.18) | 1.00 | 1.11 (0.97, 1.26) | 1.15 (0.99, 1.34) | 1.38 (1.17, 1.62) | 0.146 |
| Exclusion of participants with follow-up <2 years | 1.32 (1.16, 1.49) | 1.04 (0.92, 1.18) | 1.00 | 1.13 (1.01, 1.27) | 1.26 (1.11, 1.44) | 1.47 (1.29, 1.69) | 0.007 |
| Further adjustment for competing risk of non-COPD death | 1.32 (1.16, 1.49) | 1.04 (0.93, 1.17) | 1.00 | 1.11 (0.99, 1.25) | 1.24 (1.09, 1.40) | 1.44 (1.26, 1.65) | 0.024 |
| Definition of COPD using LLN criterion at baseline | 1.32 (1.17, 1.50) | 1.03 (0.91, 1.16) | 1.00 | 1.12 (1.00, 1.26) | 1.26 (1.11, 1.43) | 1.44 (1.26, 1.65) | 0.013 |

Note: Data were presented as hazard ratios (95% CIs). Model 3 was adjusted for education, marital status, early adulthood BMI, and lifestyle factors (smoking, alcohol consumption, physical activity, and dietary habits), stratified by age and region. Former smokers who quitted because of illness were classified as current amokers. LLN: lower limit of normal.

# Table S6. Sensitivity analysis for the association between early adulthood BMI and COPD.

| Early adulthood BMI (kg/m^2^) | <18.5 | 18.5~23.9 | 24.0~27.9 | ≥28.0 | *P*_linear_ |
| --- | --- | --- | --- | --- | --- |
| Males |  |  |  |  |  |
| Model 3 | 1.32 (1.15, 1.53) | 1.00 | 0.86 (0.78, 0.94) | 0.95 (0.73, 1.23) | <0.001 |
| Bootstrap 100 times with replacement | 1.32 (1.14, 1.54) | 1.00 | 0.86 (0.79, 0.93) | 0.95 (0.74, 1.21) | <0.001 |
| Further adjustment for household income and occupation | 1.32 (1.14, 1.53) | 1.00 | 0.86 (0.78, 0.94) | 0.94 (0.73, 1.22) | <0.001 |
| Further adjustment for passive smoking and solid fuel use in cooking and heating | 1.32 (1.14, 1.53) | 1.00 | 0.86 (0.78, 0.94) | 0.95 (0.73, 1.22) | <0.001 |
| Exclusion of participants whose weight changed≥2.5kg within 2 years | 1.25 (1.07, 1.47) | 1.00 | 0.88 (0.79, 0.97) | 0.97 (0.71, 1.33) | 0.002 |
| Exclusion of participants with follow-up of <2 years | 1.32 (1.14, 1.53) | 1.00 | 0.86 (0.78, 0.94) | 1.00 (0.78, 1.30) | <0.001 |
| Further adjustment for competing risk of non-COPD death | 1.30 (1.12, 1.50) | 1.00 | 0.85 (0.77, 0.93) | 0.91 (0.71, 1.18) | <0.001 |
| Definition of COPD using LLN criterion at baseline | 1.28 (1.11, 1.49) | 1.00 | 0.86 (0.79, 0.94) | 0.94 (0.73, 1.21) | <0.001 |
| Females |  |  |  |  |  |
| Model 3 | 1.13 (1.00, 1.28) | 1.00 | 1.01 (0.92, 1.11) | 1.17 (0.96, 1.42) | 0.900 |
| Bootstrap 100 times with replacement | 1.13 (1.00, 1.29) | 1.00 | 1.01 (0.93, 1.11) | 1.17 (0.94, 1.45) | 0.898 |
| Further adjustment for household income and occupation | 1.13 (1.00, 1.28) | 1.00 | 1.01 (0.92, 1.11) | 1.16 (0.95, 1.42) | 0.866 |
| Further adjustment for passive smoking and solid fuel use in cooking and heating | 1.13 (1.01, 1.28) | 1.00 | 1.01 (0.92, 1.11) | 1.17 (0.96, 1.42) | 0.904 |
| Exclusion of participants whose weight changed≥2.5kg within 2 years | 1.14 (0.99, 1.32) | 1.00 | 0.96 (0.86, 1.08) | 1.22 (0.97, 1.55) | 0.564 |
| Exclusion of participants with follow-up of <2 years | 1.14 (1.01, 1.29) | 1.00 | 1.03 (0.94, 1.13) | 1.19 (0.97, 1.45) | 0.852 |
| Further adjustment for competing risk of non-COPD death | 1.13 (1.00, 1.28) | 1.00 | 1.02 (0.93, 1.12) | 1.13 (0.92, 1.38) | 0.807 |
| Definition of COPD using LLN criterion at baseline | 1.13 (1.00, 1.28) | 1.00 | 1.02 (0.93, 1.12) | 1.16 (0.94, 1.42) | 0.926 |

Note: Data were presented as hazard ratios (95% CIs). Model 3 was adjusted for education, marital status, weight change, and lifestyle factors (smoking, alcohol consumption, physical activity, and dietary habits), stratified by age and region. Former smokers who quitted because of illness were classified as current amokers. LLN: lower limit of normal.

# Table S7. Sensitivity analysis for the association between early adulthood BMI and risk of COPD.

| Early adulthood BMI (kg/m^2^) | <18.5 | 18.5~24.9 | 25.0~29.9 | >=30.0 | *P_trend_* |
| --- | --- | --- | --- | --- | --- |
| Males |  |  |  |  |  |
| No. of case | 213 | 3,089 | 413 | 17 |  |
| Incidence density (/1000 person-year) | 2.55 | 2.18 | 3.25 | 3.32 |  |
| Model 1 | 1.12 (0.97, 1.29) | 1.00 | 1.06 (0.95, 1.17) | 1.67 (1.03, 2.69) | 0.534 |
| Model 2 | 1.34 (1.16, 1.55) | 1.00 | 0.89 (0.80, 0.99) | 1.11 (0.69, 1.81) | 0.001 |
| Model 3 | 1.33 (1.15, 1.54) | 1.00 | 0.89 (0.80, 1.00) | 1.16 (0.71, 1.88) | 0.001 |
| Females |  |  |  |  |  |
| No. of case | 333 | 2,313 | 468 | 40 |  |
| Incidence density (/1000 person-year) | 1.54 | 1.25 | 1.8 | 4.29 |  |
| Model 1 | 1.17 (1.04, 1.31) | 1.00 | 0.95 (0.86, 1.05) | 1.26 (0.92, 1.73) | 0.086 |
| Model 2 | 1.15 (1.02, 1.30) | 1.00 | 0.96 (0.87, 1.07) | 1.30 (0.94, 1.80) | 0.243 |
| Model 3 | 1.13 (1.00, 1.27) | 1.00 | 0.96 (0.87, 1.07) | 1.25 (0.90, 1.72) | 0.276 |
| Note: Data were presented as hazard ratios (95% CIs) unless otherwise stated. In Model 1, hazard ratios were adjusted for education and marital status; Model 2 were further adjusted for weight change based on Model 1; In Model 3, four lifestyle factors (smoking, alcohol consumption, physical activity, and dietary habits) were further included. Former smokers who quitted because of illness were classified as current amokers. The analyses were stratified according to age and region. The *P* value for sex interaction was 0.748 in Model 3. | | | | | |

# Table S8. Sensitivity analysis for the joint association of early adulthood BMI and weight change with COPD risk.

| Early adulthood BMI (kg/m^2^) | Weight change (kg) | Males | |  | Females | |
| --- | --- | --- | --- | --- | --- | --- |
|  |  | No. of cases | HR (95% CI) |  | No. of cases | HR (95% CI) |
| <18.5 | ≤-0.1 | 35 | 1.77 (1.26, 2.49) |  | 25 | 1.52 (1.01, 2.27) |
|  | 0~4.9 | 46 | 1.32 (0.98, 1.78) |  | 54 | 1.39 (1.05, 1.84) |
|  | 5.0~9.9 | 34 | 1.01 (0.71, 1.42) |  | 92 | 1.46 (1.17, 1.83) |
|  | ≥10.0 | 98 | 1.09 (0.88, 1.35) |  | 162 | 1.24 (1.04, 1.48) |
| 18.5~24.9 | ≤-0.1 | 1,506 | 1.21 (1.10, 1.33) |  | 742 | 1.17 (1.04, 1.31) |
|  | 0~4.9 | 629 | 1.00 |  | 492 | 1.00 |
|  | 5.0~9.9 | 427 | 0.88 (0.77, 0.99) |  | 464 | 1.07 (0.95, 1.22) |
|  | ≥10.0 | 527 | 0.91 (0.81, 1.03) |  | 615 | 1.37 (1.22, 1.55) |
| 25.0~29.9 | ≤-0.1 | 347 | 1.18 (1.04, 1.35) |  | 328 | 1.09 (0.94, 1.25) |
|  | 0~4.9 | 34 | 0.76 (0.54, 1.07) |  | 58 | 0.83 (0.63, 1.09) |
|  | 5.0~9.9 | 15 | 0.71 (0.42, 1.18) |  | 42 | 1.19 (0.87, 1.63) |
|  | ≥10.0 | 17 | 1.14 (0.71, 1.85) |  | 40 | 1.53 (1.11, 2.12) |
| ≥30.0 | ≤-0.1 | 12 | 1.32 (0.74, 2.33) |  | 36 | 1.34 (0.95, 1.89) |
|  | 0~4.9 | 2 | 5.09 (1.26, 20.50) |  | 2 | 0.94 (0.23, 3.79) |
|  | 5.0~9.9 | 2 | 12.41 (3.05, 50.44) |  | 0 | - |
|  | ≥10.0 | 1 | 4.50 (0.63, 32.23) |  | 2 | 11.07 (2.75, 44.62) |
| Note: Hazard ratios were estimated by deriving the 4-by-4 composite exposure of early adulthood BMI and weight change. Results were based on Model 3, with adjustment for education, marital status, and lifestyle factors (smoking, alcohol consumption, physical activity, and dietary habits), stratified by age and region. Former smokers who quitted because of illness were classified as current amokers. *P* values for interaction were 0.097 in males and 0.014 in females. | | | | | | |
